# A Rare Presentation of Cytomegalovirus Mononucleosis in a Nine-Year-Old Girl: A Case Report

**DOI:** 10.7759/cureus.104242

**Published:** 2026-02-25

**Authors:** Sabi Rana, Sailuja Maharjan, Bikram Babu Karki, Reham Bukhari, Sushant Dhungel

**Affiliations:** 1 Otolaryngology-Head and Neck Surgery, Kulhudhuffushi Regional Hospital, Kulhudhuffushi, MDV; 2 Pathology, Kulhudhuffushi Regional Hospital, Kulhudhuffushi, MDV; 3 Pathology, Baidya &amp; Banskota (B&amp;B) Hospital, Lalitpur, NPL; 4 Pediatrics, Kulhudhuffushi Regional Hospital, Kulhudhuffushi, MDV; 5 Internal Medicine, Kulhudhuffushi Regional Hospital, Kulhudhuffushi, MDV

**Keywords:** atypical lymphocytosis, cytomegalovirus, ebstein-barr virus, infectious mononucleosis, mononucleosis syndrome, serology

## Abstract

Epstein-Barr virus (EBV) and cytomegalovirus (CMV) are common viruses from the Herpesviridae family. While EBV is the classic cause of infectious mononucleosis (IMN), characterized by fever, pharyngitis, cervical lymphadenopathy, and atypical lymphocytes, CMV can also cause a similar mononucleosis syndrome, though it is less frequently reported. Mononucleosis caused by both EBV and CMV is more common in preadolescent children or young adults. Here, we present a case of a nine-year-old girl presenting with a two-week history of sore throat, fever, cervical lymphadenopathy, and cough. Additional findings included periorbital swelling and splenomegaly. Peripheral blood smear revealed atypical lymphocytosis, prompting serologic evaluation for EBV and CMV. IgM antibodies for EBV were negative, whereas both IgM and IgG antibodies for CMV tested positive, thus indicating an active infection, which is either a primary (first-time) infection or a reactivation of a previous infection. She recovered with conservative management gradually over eight days. This case highlights that although uncommon, CMV mononucleosis should be considered in patients presenting with fever, sore throat, and cervical lymphadenopathy. Additional findings may include periorbital edema and splenomegaly. Diagnosis relies on atypical lymphocytosis and serologic testing, and management is generally supportive. Antiviral therapy and corticosteroids are reserved for severe disease in immunocompromised patients.

## Introduction

Infectious mononucleosis (IMN) is a viral infection presenting with a classical triad of fever, pharyngitis, and lymphadenopathy [1]. Although Epstein-Barr virus (EBV) and cytomegalovirus (CMV) infect a large proportion of the global population-reaching seroprevalence rates of 60% to 100% among adults-clinically apparent IMN most commonly affects adolescents and young adults, particularly in developed countries [2]. It primarily affects the reticuloendothelial and lymphatic system [3]. The condition is most commonly caused by the EBV, with CMV accounting for up to 10% of cases [4]. Infections caused by CMV often present with symptoms indistinguishable from those caused by EBV [5]. However, CMV mononucleosis often presents with milder or absent pharyngitis, less prominent lymphadenopathy, and prolonged low-grade fever, though features such as hepatosplenomegaly, abdominal pain, periorbital edema, and atypical lymphocytosis may still occur. However, the similarity complicates clinical diagnosis because the two infections cannot be reliably differentiated based on symptoms and physical examination alone. As a result, laboratory testing is necessary to confirm the underlying cause. Rarely, IMN can be caused by other agents like human herpesvirus 6 (HHV-6), HIV, adenovirus, or *Toxoplasma gondii*, making it difficult to identify the exact etiology without specific assays.

Immunoassays detecting viral capsid antigen (VCA) immunoglobulin M (IgM), VCA immunoglobulin G (IgG), and Epstein-Barr nuclear antigen-1 (EBNA-1) IgG are routinely performed to confirm the diagnosis of IMN caused by EBV [6]. These markers are not indicators of CMV infection [7]. Heterophile antibody (Ab) tests are positive in only 25%-50% of children under 12 years of age and are usually negative in cases caused by CMV, HHV-6, or *Toxoplasma* [3], underscoring a key diagnostic limitation and highlighting the importance of pathogen-specific serologic testing-particularly CMV-specific assays, when heterophile results are negative but clinical suspicion remains high.

Atypical lymphocytes play a key role in supporting a viral etiology in IMN. These cells are activated cytotoxic T lymphocytes responding to infected B cells. The presence of a significant number of atypical lymphocytes (typically >10% of total lymphocytes) is a cornerstone in the diagnosis of IMN.

IMN is generally a self-limiting condition; therefore, supportive care is the mainstay of treatment. However, it can be clinically significant due to complications such as splenic enlargement with risk of rupture, airway obstruction from severe tonsillar hypertrophy, hepatitis, hematologic abnormalities, and, rarely, neurologic or cardiac involvement-highlighting the importance of accurate diagnosis and appropriate monitoring.

For most people, the acute symptoms of IMN (mono) last for two to four weeks. Antiviral therapy has a limited impact on the clinical features or disease progression [8], and corticosteroid therapy is reserved for complications such as airway obstruction, severe hemolysis, or thrombocytopenia [3]. Close monitoring of CMV mononucleosis is particularly important in immunocompromised individuals (such as transplant recipients, patients with HIV/AIDS, or those receiving immunosuppressive therapy) and in pregnant women, due to the higher risk of severe disease, systemic complications, or congenital transmission.

## Case presentation

We present a case of a nine-year-old girl who presented to our ENT outpatient department with a two-week history of gradual onset of throat pain, which was persistent throughout the illness. On the same day, she reported low- to moderate-grade fever, often ranging from 38°C to 38.9°C, and the pattern was intermittent. Within a few days of symptom onset, she developed a productive cough with yellow sputum associated with nasal obstruction and nasal discharge. Her past medical and surgical history was unremarkable.

On physical examination, the child appeared ill-looking but was alert and conscious, with a pulse rate of 86 bpm, respiratory rate of 30/min, SpO_2_ of 95% in room air, and temperature of 38.8°C. She had bilateral enlarged grade III congested tonsils with exudates. Bilateral jugulodigastric lymph nodes were palpable, approximately 3 x 3 cm^2^, tender on palpation, freely mobile, with healthy overlying skin. Chest examination revealed bilateral decreased air entry with wheezes on auscultation.

The patient was admitted, hematological and inflammatory parameter investigations (Table 1) were sent, and symptomatic management was commenced. Her total leukocyte count (TLC) was raised to 18.4 × 10^3^/µL with lymphocyte predominance. Erythrocyte sedimentation rate (ESR) was raised to 18 mm/1st hour, and C-reactive protein (CRP) was 13.5 mg/L.

**Table 1:** Hematological and inflammatory parameters on admission showing raised total leukocyte count (TLC) with lymphocyte predominance and raised ESR and CRP ESR: erythrocyte sedimentation rate; CRP: C-reactive protein; µL: microliter; g/dL: grams per deciliter; mm/1st hour: millimeters in the first hour; mg/L: milligrams per liter

	Result	Unit	Reference range
Total leukocyte count	18.16	×10^3^/µL	4.27-11.40
Differential leukocyte count			
Neutrophil	26	%	29.8-71.4
Lymphocyte	61	%	16.7-57.8
Monocyte	0.5	%	4.2-11.3
Eosinophil	0	%	0-4
Basophil	0.5	%	0-0.6
Hemoglobin	9.9	g/dL	10.6-13.2
Platelet count	199	×10^3^/µL	150-450
ESR	18	mm/1st hour	2-15
CRP	13.5	mg/L	<10

**Figure 1:**
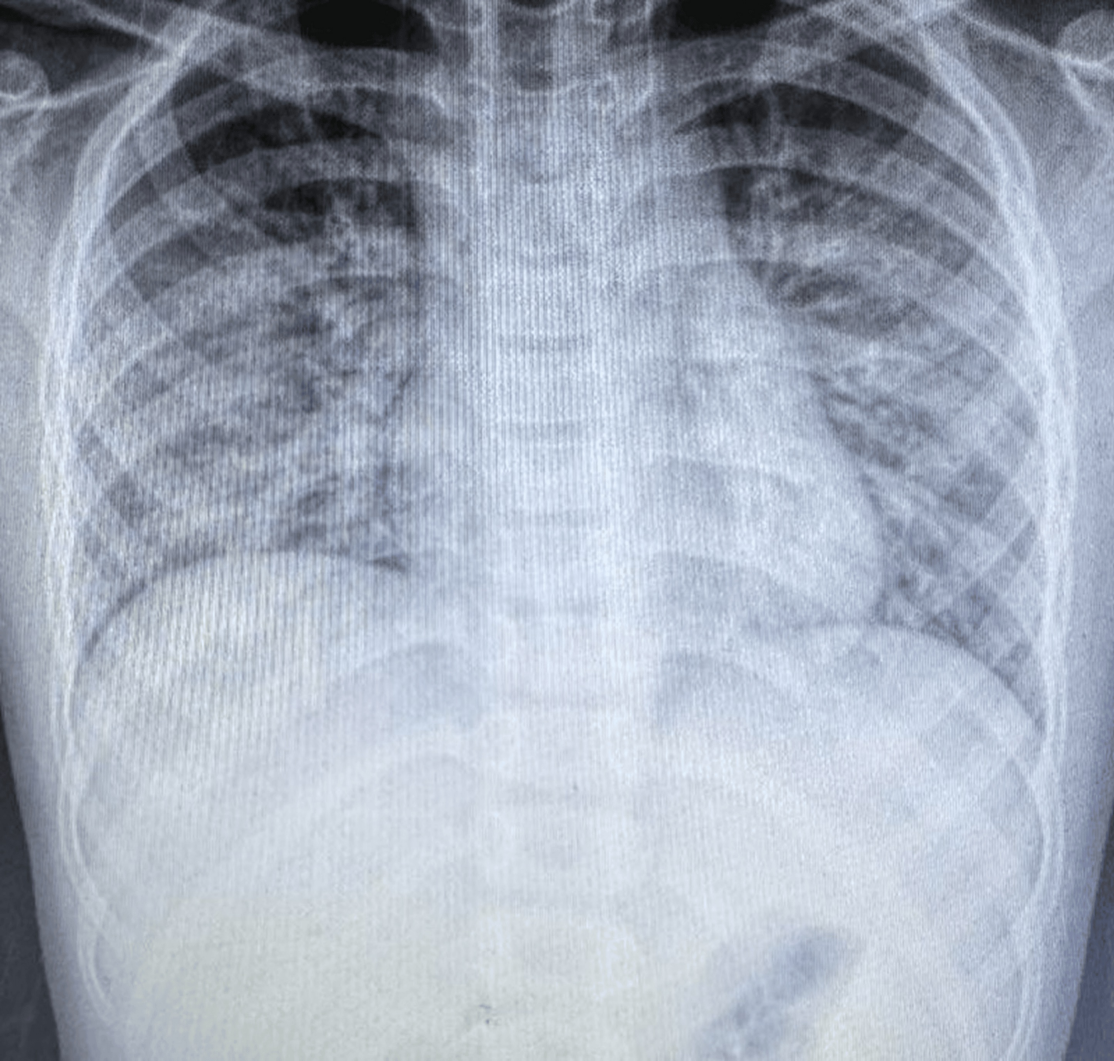
Chest X-ray PA view showing diffuse bilateral opacities PA: posteroanterior

Chest X-ray (Figure 1) showed diffuse bilateral opacities more pronounced in the lower lobes, suggestive of pneumonia. Ultrasound of the neck revealed bilateral multiple cervical lymphadenopathy (largest on the right submandibular region: 3 × 3.1 cm^2^)-some oval, others round, some with preserved fatty hilum-suspecting an inflammatory nature. Both of these were done on the same day of admission.

During admission, the patient developed bilateral periorbital edema without associated rash, redness, or ocular discharge. She was admitted during the day, and by the evening, the swelling had become clearly noticeable. She also complained of abdominal pain with three episodes of vomiting the next day. Ultrasonography of the abdomen and pelvis showed mild splenomegaly (12.2 cm and homogenous texture) and porta hepatic lymphadenopathy, which was not palpable clinically. The liver and bilateral kidneys appeared to be normal.

Her liver function test (Table 2) was done on the second day of admission, which showed raised liver enzymes, including both total and direct bilirubin, serum glutamic-oxaloacetic transaminase (SGOT), serum glutamic-pyruvic transaminase (SGPT), gamma-glutamyl transferase (GGT), and lactate dehydrogenase (LDH), suggestive of hepatitis.

**Table 2:** Liver function test: abnormal result with elevated bilirubin, SGOT, SGPT, GGT, and LDH levels mg/dL: micrograms per deciliter; g/dL: grams per deciliter; U/L: units per liter

	Result	Unit	Reference range
Total bilirubin	2.2	mg/dL	0.2-1.3
Direct bilirubin	1.5	mg/dL	0.0-0.3
Total serum protein	6	g/dL	6.3-8.2
Albumin/globulin ratio	1.2		
Serum glutamic-oxaloacetic transaminase (SGOT) test	153	U/L	14-36
Serum glutamic-pyruvic transaminase (SGPT) test	341	U/L	<35
Gamma-glutamyl transferase (GGT)	247	U/L	2-43
Lactate dehydrogenase (LDH) test	785	U/L	120-246

The throat swab culture was negative for group A *Streptococcus*. Given that the incubation period for streptococcal pharyngitis is typically 2-5 days and her symptoms had persisted for two weeks, this finding supports a viral etiology. Blood culture and sputum culture did not show any growth after 72 hours of incubation.

**Figure 2:**
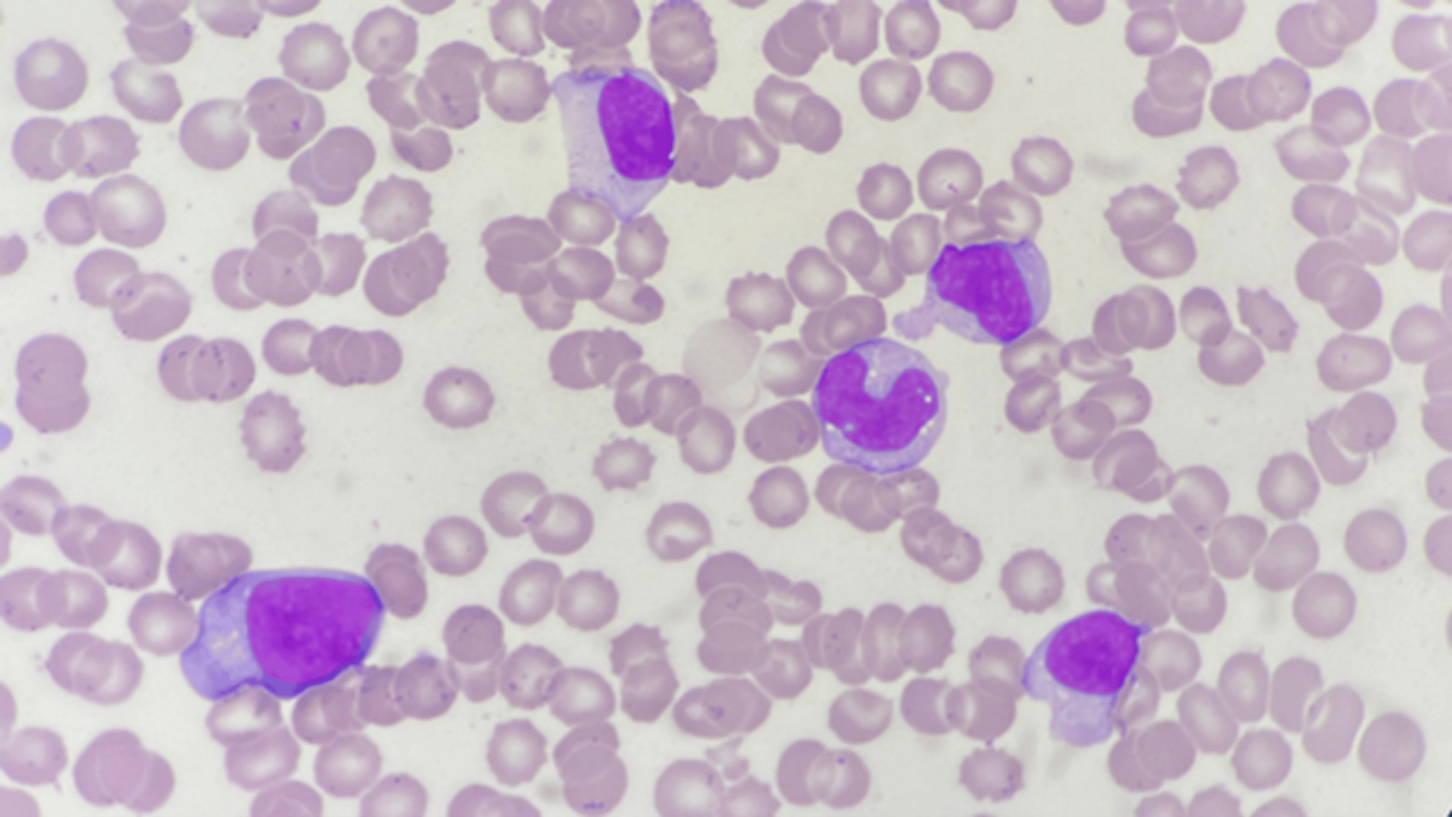
Peripheral blood smear showing atypical lymphocytes with eccentric nucleus, conspicuous nucleoli, deep blue cytoplasm with the cell membrane abutting the adjacent RBCs (x100 HPF) HPF: high-power field

Peripheral blood smear (PBS) examination showed absolute lymphocytosis with the presence of 12% atypical lymphocytes (Figure 2). These atypical lymphocytes were large in size with an eccentric nucleus and conspicuous nucleoli. The cytoplasm was deep blue, moderate in amount, with the cell membrane abutting the adjacent RBCs. These findings, in conjunction with the clinical features, raised the possibility of viral infections like EBV and CMV. Following this, the viral serological markers were tested.

Following this, her viral serological markers were tested (Tables 3, 4).

**Table 3:** CMV serology with positive IgM and IgG antibody CMV: cytomegalovirus; IgG: immunoglobulin G test; IgM: immunoglobulin M test; AU/mL: arbitrary units per milliliter

CMV	Result	Reference range
IgG	>250 AU/mL	0.0-6.0
IgM	1.00 index	0.00-0.85

**Table 4:** EBV serology: negative IgM antibody EBV: Ebstein-Barr virus; IgM: immunoglobulin M test

EBV	Result	
IgM	2.76	<9: negative
		9-11: indeterminant
		>11: positive

Screening for hepatitis B and C along with HIV was also negative. Additionally, our patient did not exhibit clinical features typically associated with these infections, such as jaundice or marked constitutional symptoms (loss of appetite, weight loss, and night sweats) suggestive of viral hepatitis, nor risk factors or systemic manifestations consistent with acute HIV seroconversion illness, ruling out these infections. Although her symptoms-including purulent sputum, high-grade fever, and respiratory findings-suggested a possible bacterial co-infection, laboratory and serologic investigations confirmed a viral etiology.

The final diagnosis was thus confirmed to be CMV mononucleosis with positive IgG and IgM Ab for CMV (Table 3), which generally indicates an active infection, which is either a primary (first-time) infection or a reactivation of a previous infection. The patient was managed conservatively with antipyretics and adequate hydration. Respiratory symptoms were closely monitored. Her TLC began declining steadily from the third day of admission, and symptoms resolved completely over eight days. She was discharged following full recovery with safety net advice and asked to follow up after two weeks. On follow-up after two weeks, her total and differential leukocyte count, CRP, and liver function test all came within normal limits, and PBS showed normal WBCs in morphology without any atypical lymphocytes.

## Discussion

CMV, a member of the Herpesviridae family, is a ubiquitous virus transmitted mostly by contact with infectious body fluids (saliva, urine, and genital) or by the transplacental route [9]. Congenital CMV infection is a well-established risk factor for permanent childhood hearing loss. Primary CMV infection in immunocompetent children is often asymptomatic or may present with nonspecific viral symptoms. CMV accounts for up to 10% of cases of mononucleosis-like illness [4] closely resembling EBV mononucleosis. Although most cases of CMV mononucleosis are self-limiting, hepatitis with elevated aminotransferases and atypical lymphocytosis are commonly reported features, and a range of severe complications, including Guillain-Barré syndrome, meningoencephalitis, myocarditis, thrombocytopenic purpura, vasculitis, vascular thrombosis, pneumonia, and colitis, have been described even in immunocompetent patients, although they are rare [10].

The child in the presented case initially showed classic signs of tonsillitis and cervical lymphadenitis, which are common in many upper respiratory tract infections. However, the concurrent presence of respiratory symptoms, radiological evidence of pneumonia, and splenomegaly added complexity to the clinical picture. Such presentations often lead to suspicion of viral etiology. Negative throat swab and blood culture and atypical lymphocytes in PBS further raised the possibility of viral etiology, including CMV, which was subsequently confirmed by positive CMV-specific IgM and IgG serology.

According to a study by Mohan et al., the classical symptoms of mononucleosis (sore throat, cervical lymphadenopathy, and tonsil enlargement) were significantly less common in CMV cases, but there was a prolonged duration of fever in CMV mononucleosis as compared to EBV mononucleosis [11]. In another study by Medović et al., a greater percentage of children with CMV mononucleosis had symptoms such as stomach pain due to mesenteric lymph node enlargement and/or liver/spleen enlargement, as well as eyelid swelling due to impaired lymphatic drainage from the facial and eye area [12]. Notably, our patient also developed periorbital swelling and mild splenomegaly, findings that are consistent with the described clinical spectrum of CMV mononucleosis.

Around 92% of patients with acute CMV infection had elevation of liver transaminase levels in a study by Medović et al., which is similar to our case, where SGOT was 153 U/L and SGPT was 341 U/L. In contrast to other viral causes of hepatitis, CMV-infected patients are anicteric, and their aspartate and alanine transaminase levels are rarely above five times the normal range [13]. Pulmonary manifestations of CMV disease are rare (8%) in immunocompetent patients [14]. CMV pneumonia, if present, usually manifests with hypoxemia, respiratory failure, and diffuse interstitial infiltrates on chest X-ray. In patients with otherwise unexplained lymphocytosis and increased serum transaminases, one should consider the possibility of CMV pneumonia even in immunocompetent patients [15], as in our patient.

Any febrile illness in which more than 10% of the patient’s lymphocytes are atypical should raise the suspicion of mononucleosis [3]. These atypical cells consist of Downey type II cells (larger cells with abundant pale cytoplasm) and Downey type III cells (large cells with one or more prominent nucleoli and abundant deeply basophilic cytoplasm) [16]. The presence of atypical lymphocytes may mimic blast cells in PBS and could raise the possibility of hematolymphoid neoplasm, further requiring bone marrow examination. The heterophile Ab test is a rapid screening test used to support the diagnosis of EBV mononucleosis. In contrast, this test is usually negative in CMV mononucleosis. Although rare, CMV infection, serum sickness, or other viral illness may occasionally result in a false-positive heterophile Ab test [3].

Thus, the most reliable diagnostic test for confirming CMV mononucleosis is serologic testing for CMV-specific IgM Abs, which are typically positive in the majority of patients during the symptomatic phase of the illness. Also, serial monitoring of CMV IgG titers is considered reliable for determining whether CMV is the underlying cause of fever as CMV-specific IgG Ab levels demonstrate at least a fourfold rise during the acute phase [3].

Most cases of CMV mononucleosis improve without any specific antiviral treatment. Targeted antiviral therapy with ganciclovir or valganciclovir is generally reserved for severe cases or for immunocompromised patients [9]. Primary CMV infection can occasionally present with a protracted clinical course of more than six weeks in otherwise immunocompetent patients before spontaneous recovery occurs [10]. Our patient’s gradual improvement with supportive care alone aligns with the natural course of uncomplicated CMV infection.

## Conclusions

Symptomatic CMV mononucleosis in children is rare, as most CMV infections in this age group are asymptomatic or present with mild, nonspecific symptoms. This case thus highlights the importance of considering CMV infection in children presenting with prolonged fever, lymphadenopathy, atypical lymphocytosis, and elevated liver enzymes-especially when EBV serology is negative. CMV mononucleosis can mimic EBV infection but may also present with additional features such as periorbital edema, abdominal pain, and hepatosplenomegaly, as observed in our patient. Although pulmonary involvement is rare in immunocompetent individuals, clinicians should remain vigilant when respiratory symptoms are present, as our patient did develop findings suggestive of pneumonia. PBS with atypical lymphocytes aids in diagnosis, while serologic testing remains the most reliable diagnostic tool with IgM Ab positivity of CMV indicating active infection. Most cases, including ours, resolve with supportive care alone, emphasizing the self-limiting nature of CMV mononucleosis in immunocompetent children.
